# Inducible knock-down of *GNOM* during root formation reveals tissue-specific response to auxin transport and its modulation of local auxin biosynthesis

**DOI:** 10.1093/jxb/ert475

**Published:** 2014-01-22

**Authors:** Jingzhe Guo, Jun Wei, Jian Xu, Meng-Xiang Sun

**Affiliations:** ^1^Department of Cell and Developmental Biology, College of Life Science and State Key Laboratory of Hybrid Rice, Wuhan University, Wuhan 430072, China; ^2^Department of Biological Sciences and NUS Centre for BioImaging Sciences, National University of Singapore, Science Drive 4, Singapore117543

**Keywords:** *Arabidopsis*, auxin biosynthesis, auxin transport, *GNOM*, inducible gene expression, root.

## Abstract

In plants, active transport of auxin plays an essential role in root development. Localization of the PIN1 auxin transporters to the basal membrane of cells directs auxin flow and depends on the trafficking mediator GNOM. GNOM-dependent auxin transport is vital for root development and thus offers a useful tool for the investigation of a possible tissue-specific response to dynamic auxin transport. To avoid pleiotropic effects, DEX-inducible expression of *GNOM* antisense RNA was used to disrupt *GNOM* expression transiently or persistently during embryonic root development. It was found that the elongation zone and the pericycle layer are the most sensitive to GNOM-dependent auxin transport variations, which is shown by the phenotypes in cell elongation and the initiation of lateral root primordia, respectively. This suggests that auxin dynamics is critical to cell differentiation and cell fate transition, but not to cell division. The results also reveal that GNOM-dependent auxin transport could affect local auxin biosynthesis. This suggests that local auxin biosynthesis may also contribute to the establishment of GNOM-dependent auxin gradients in specific tissues, and that auxin transport and local auxin biosynthesis may function together in the regulatory network for initiation and development of lateral root primordia. Thus, the data reveal a tissue-specific response to auxin transport and modulation of local auxin biosynthesis by auxin transport.

## Summary

Using DEX-inducible expression of *GNOM* antisense RNA, it was shown that GNOM-dependent auxin transport could affect local auxin biosynthesis, and this may also contribute to the establishment of GNOM-dependent auxin gradients.

## Introduction

In *Arabidopsis* and other plants with taproot systems, the root forms during embryogenesis and develops during seed germination; lateral roots are then generated from the main root. One of the key players regulating these processes is the plant hormone auxin, whose homeostasis is established and maintained by both polar transport (Benkova *et al.*, 2003) and local biosynthesis (Zhao *et al.*, 2001; Cheng *et al.*, 2006, 2007; Stepanova *et al.*, 2008; Tao *et al.*, 2008).

Active polar auxin transport moves auxin in the root tip. Experimental evidence and mathematical modelling have shown that auxin at the tip of the primary root is transported by PINFORMED (PIN) auxin efflux proteins, thus maintaining post-embryonic primary root growth (Blilou *et al.*, 2005; Grieneisen *et al.*, 2007). Indeed, combinations of multiple mutants of *PIN* genes, such as *pin1 pin3 pin4* and *pin1 pin3 pin4 pin7*, produce very short primary roots (Benkova *et al.*, 2003). PIN family proteins are constantly recycled between the plasma membrane and endosomal compartments through vesicle trafficking (Anders *et al.*, 2008). The ARF-GEF (guanine nucleotide exchange factor of ADP-ribosylation factor) protein GNOM mediates this trafficking (Steinmann *et al.*, 1999; Geldner *et al.*, 2001). GNOM is required for basal localization of the auxin efflux carrier protein PIN1, acting by mediating endosome–plasma membrane recycling (Geldner *et al.*, 2001, 2003) and GNOM-dependent PIN1 transcytosis to the basal cell membrane (Kleine-Vehn *et al.*, 2008). *gnom* mutants show defects in apical and basal pole specification during embryogenesis and fail to produce viable seedlings (Mayer *et al.*, 1996). Weak *gnom* mutants produce viable seedlings, but fail to maintain root meristem activity and quiescent centre (QC) cell identity, suggesting a requirement for GNOM during root formation (Geldner *et al.*, 2004). Interestingly, a recent study of tissue-specific expression of *GNOM* in embryos further revealed that GNOM might play different roles in different tissues (Vroemen *et al.*, 1996; Wolters *et al.*, 2011). Thus, it is also of interest to unravel the tissue-specific response to GNOM-dependent auxin transport, as part of the auxin regulatory network, during post-embryonic root development.

Auxin transport is not the only mechanism required for root development. Root tip cells also have the capacity to synthesize auxin locally (Petersson *et al.*, 2009), and during embryogenesis *YUCCA*-mediated auxin biosynthesis is important for specification of the embryonic root meristem (Cheng *et al.*, 2007). Together, these data suggest that primary root formation and development depend on both auxin transport and auxin biosynthesis. As for development of lateral roots, available evidence also indicates the involvement of both auxin transport and auxin biosynthesis. For example, polar auxin transport mutants such as *pin1*, *pin2*, *pgp4* (Benkova *et al.*, 2003; Terasaka *et al.*, 2005; Wu and Lewis, 2007; Mravec *et al.*, 2008), and *aux1* (De Smet *et al.*, 2007) also show lateral root defects. Studies on these mutants and further analysis of auxin transport-related proteins showed that spatial–temporal expression and localization of auxin transport components are required for both initiation and development of lateral root primordia (LRPs), substantiating a role for polar auxin transport in lateral root formation (Benkova *et al.*, 2003; Wu and Lewis, 2007). Locally induced auxin biosynthesis in a single pericycle cell is sufficient to initiate LRPs (Dubrovsky *et al.*, 2008). Also, lateral root formation was inhibited in a *spl-D* (*SPOROCYTELESS* dominant) mutant due to suppression of expression of the auxin synthesis genes *YUC2* and *YUC6* (Li *et al.*, 2008). Notably, the auxin biosynthesis gene *ASA1* was found to regulate auxin transport during lateral root formation in jasmonic acid-treated seedlings (Sun *et al.*, 2009), and ethylene appears to stimulate auxin biosynthesis and increase auxin transport (Ruzicka *et al.*, 2007; Stepanova *et al.*, 2007; Swarup *et al.*, 2007). These data suggest that both auxin transport and biosynthesis are involved in root formation and that auxin biosynthesis may contribute to the regulation of auxin transport, although the regulatory relationships still remain to be clarified.

However, despite these recent advances, the questions of whether auxin transport could, in turn, regulate auxin biosynthesis to achieve auxin homeostasis and how auxin transport and auxin biosynthesis contribute directly to root system development remain largely unanswered. This is partly due to the fact that most of our knowledge was obtained by mutant analysis of auxin-related genes at post-embryonic stages or by treatment with polar auxin transport inhibitors. Because mutants of auxin transport or auxin synthesis generally show defects in early embryogenesis (Mayer *et al.*, 1996; Benkova *et al.*, 2003; Cheng *et al.*, 2007), the phenotypes observed in mutant seedlings might be cumulative or indirect effects. Conditionally blocking polar auxin transport at specific development stages with chemical inhibitors could avoid such effects, but often causes other non-specific phenotypes. To overcome these issues, more focused approaches have been developed to unravel the primary roles of both auxin transport and biosynthesis. For example, constitutive tissue-specific or inducible gene expression systems have been developed and used to identify the AGC protein kinases PINOID (PID), WAG1, and WAG2 as novel determinants of PIN polarity and polar auxin transport (Friml *et al.*, 2004; Dhonukshe *et al.*, 2010). Also, auxin overproduction in the QC (Blilou *et al.*, 2005) and examination of clonal sectors of wild-type roots (Fischer *et al.*, 2006) resulted in the discovery of an auxin reflux loop in the root tip and mechanisms driving root hair planar polarity. Thus, inducible expression of key auxin transport- or auxin biosynthesis-related genes will allow auxin dynamics to be controlled at the cellular level and the examination of possible differential responses of root cells to transient auxin dynamics at unprecedented spatial–temporal resolution. In addition, such an inducible gene expression system will facilitate observation of the tissue-specific response to GNOM-dependent auxin transport at a moderated level during root development.

Here, the development of a dexamethasone- (DEX) inducible antisense system in *Arabidopsis* is reported which allowed *GNOM* expression to be knocked down, in a reversible manner. Based on the investigation of root development in response to transient or continuous disruption of auxin transport, it was found that root responds to GNOM-mediated auxin transport in a tissue-specific manner. It was also found that auxin biosynthesis and auxin transport may have a combinatorial effect on lateral root initiation and development.

## Materials and methods

### Plant materials, growth conditions, and transformation

All transgenic *Arabidopsis thaliana* were in the ecotype Columbia-0 background. DR5::GUS (β-glucuronidase) (Ulmasov *et al.*, 1997), pPIN1::PIN1-GFP (green fluorescent protein) (Xu *et al.*, 2006), pPIN2::PIN2-GFP (Xu and Scheres, 2005), pPIN3::PIN3-EGFP (Blilou *et al.*, 2005), pPIN7::PIN7-EGFP (Laskowski *et al.*, 2008), pWOX5:YFP (yellow fluorescent protein) (Blilou *et al.*, 2005), DR5*rev*::GFP (Benkova *et al.*, 2003), and CYCB1;1::GUS (Colon-Carmona *et al.*, 1999) have been described previously.

For plant transformation, Col-0 seeds were germinated and grown in 9cm pots containing moistened vermiculite in the greenhouse at 22±1 °C with a 16/8h (light/dark) photoperiod and 60% relative humidity. First bolts were cut to encourage proliferation of secondary bolts and, after 4–6 d, inflorescences with flowers were used for plant transformation. For transformation, constructs were introduced into *Agrobacterium tumefaciens* strain LBA4404. *Arabidopsis* plants with flowering secondary bolts were transformed with *A. tumefaciens* by the floral dip method (Clough and Bent, 1998). Transgenic plants were selected on Murashige and Skoog (MS) medium, which contained 20mg l^–1^ hygromycin (Roche).

Seeds were surface sterilized in 4% sodium hypochlorite, washed three times with sterile water, stratified for 3 d at 4 °C, and then germinated on plates containing 0.5× MS (Murashige and Skoog, 1962) basal salt mixture, 1% (w/v) sucrose, 3mM MES (Sigma-Aldrich), 0.4% (w/v) phytagel (Sigma-Aldrich), and 0.2% (w/v) agar powder (pH 5.7). Square dishes were used and placed vertically at 22 °C, with a 16h light/8h dark cycle.

### DNA constructs

A 738bp region of the *GNOM* sequence, which contains the region from position +1 to +738 downstream of the translation initiation site (ATG), was amplified from wild-type *Arabidopsis* genomic DNA by Pyrobest DNA polymerase (TaKaRa) with the forward primer 5′-TAG*ACTAGT*ATGGGTCGCCTAAAG-3′ and reverse primer 5′-TGA*CTCGAG*CTCTTGTTTGATGCT-3′. The underlined nucleotides correspond to the *Spe*I and *Xho*I restriction sites, respectively. The PCR product was cloned into the pGEM-T Easy vector (Promega). Then the insert sequence was confirmed by DNA sequencing. The correct clone was inserted in reverse orientation via the *Spe*I–*Xho*I sites into vector pTA211 (Aoyama and Chua, 1997) downstream of 6× UAS, which harbours amplified DNA fragments encoding a GAL4-VP16–rat glucocorticoid-binding domain construct between the G1090 promoter and the pea (*Pisum sativum*) rbcs-E9 terminator. For a control, the empty vector was directly transformed into *Arabidopsis*.

### Dexamethasone induction of antisense expression

A stock solution of DEX (Sigma-Aldrich) dissolved in ethanol was added to the growth medium at 30 μM DEX and 0.1% (v/v) ethanol (EtOH). All controls contained medium with 0.1% (v/v) EtOH. For DEX treatment, seeds were either directly germinated on inducing medium or pre-cultured for the desired number of days before being transferred to inducing medium. Parallel experiments were performed on empty vector transgenic lines with identical conditions. For the experiments on the time course of DEX induction, a group of 10–15 seedlings were manually transferred with forceps to DEX or EtOH plates. The time was recorded after the transfer of each group and seedlings were then used for analysis at the indicated time point.

### RNA isolation and quantitative real-time RT–PCR

Total RNA was extracted from roots of treated samples using TriPure Isolation Reagent (Roche). First-strand cDNAs were synthesized from 150ng of total RNA with 2.5 μM anchored-oligo(dT)_18_ primer (Roche) and 2.5 μM random hexamer primer (Roche) using a Transcriptor First Strand cDNA Synthesis Kit (Roche). Reverse transcription was carried out at 55 °C for 60min, and reverse transcriptase was inactivated by heating to 85 °C for 5min. For detection of induced antisense *GNOM* transcripts, 150ng of total RNA was transcribed by Transcriptor Reverse Transcriptase (Roche) primed with a mixture of gene-specific reverse transcription primers (500nM each) of reference genes and the antisense *GNOM* fragment (Supplementary Table S1 available at *JXB* online). The reverse transcription reaction was first incubated at 25 °C, followed by a 60min incubation at 50 °C, and then heated to 85 °C for 5min to inactivate reverse transcriptase. All quantitative PCR (qPCR) primers (Supplementary Table S2 available at *JXB* online) for each examined gene were synthesized by Invitrogen, and they were designed either to span an intron or to bridge an exon–exon junction to discriminate or avoid amplification from genomic DNA. qPCR analyses were performed with two technical repeats and two or three biological repeats, using FastStart Universal SYBR Green Master (Roche) on a Rotor-Gene 6000 real-time multiplexing system (Corbett Research, Australia). PCR cycling conditions for amplification were 95 °C for 10min and then 45 cycles of 95 °C for 10 s, 60 °C for 60 s. Absence of primer dimers and genomic amplification was analysed by both melting curve analysis and electrophoresis in a 3% agarose gel. PCR efficiency for each primer pair was estimated using the LinRegPCR software (Ramakers, 2003). The average efficiency of all reactions in a single run (which was always >1.90) was used for further calculation. To find the most stably expressed reference genes for normalization, qPCR was performed on cDNA of 16 samples crossing all treatments, using primers for five reference genes (*At5g25760 UBC*, *At1g13320 PP2A subunit A3*, *At4g26410 Expressed unknown protein*, *At4g34270 TIP41 like*, and *At5g46630 Clathrin adaptor complex subunit*) as reported by Czechowski (2005). Then, they were put into qbasePLUS software (Biogazelle, Belgium) for geNorm analysis (Vandesompele *et al.*, 2002). The expression of each examined gene was normalized to the geometric mean of the reference genes *TIP41 like* and *UBC*, as indicated by geNorm analysis (V_2/3_ <0.15). Finally, relative expression data of all examined genes were automatically generated by qbasePLUS software (Version 1.5).

### Generation of InAGN9 lines with marker genes

InAGN9 lines with different marker genes were produced by genetic crossing between InAGN9 and all the other marker lines. F_1_ generation seeds from each of the crosses were planted and F_2_ seeds were collected after self-pollination. These F_2_ seeds were then germinated on MS medium supplemented with 30 μM DEX for 5 d and the seedlings with short roots were examined under a fluorescent microscope for the expression of the marker genes. Those seedlings with both short roots and expression of marker genes were planted into soil and F_3_ seeds were collected individually from each of the plants. Finally, these F_3_ seeds were germinated again on MS medium supplemented with 30 μM DEX for 5 d and seeds from those lines where all seedlings showed both a consistent phenotype of short roots and the expression of marker genes were considered as double homozygous seeds for both loci.

### Root assays

For root growth curve assays, transgenic *Arabidopsis* were grown in vertically positioned square dishes and the position of each root tip was recorded every 2 d with a marker pen. At the end of the test, all plates were scanned together with a ruler on a flat-bed scanner (Microtek) and root growth was measured by Image J 1.40 software (NIH).

The number of root meristematic cells was measured as previously described (Geldner *et al.*, 2004). The cells in the cortical cell file of the primary root tip were counted from the cortex/endodermis initial cell to the cell that shows distinct elongation.

For root cortical cell length measurement, images of cortical cells in the differentiation zone for each root were captured using an Olympus IMT-2 inverted DIC microscope equipped with a CCD camera (Cool SNAP HQ, Roper Scientific). Cell length was measured using Image J software (NIH). The cell production rate was calculated as the length of root elongated during 24h divided by the average length of cortical cells in the root region grown during the period.

Lateral roots were counted under an Olympus CK40 inverted microscope. For observation of LRPs, roots were cleared and classified according to Malamy and Benfey (1997) under an Olympus VANOX microscope. Lateral root induction by J-hook formation was carried out as described (Laskowski *et al.*, 2008). Vertically cultured roots were turned 180 ° and remained under the same conditions until observation.

### Hormone treatment

Naphthalene acetic acid (NAA; Sigma-Aldrich) and indole-3-acetic acid (IAA; Sigma-Aldrich) were dissolved with a small volume of 1 N NaOH and then diluted with water to a final concentration of 100mM before filter sterilization. These stock solutions were added together with DEX or EtOH to autoclaved medium at the indicated concentrations. Pre-cultured seedlings were transferred to the media for treatment or seeds were directly germinated on the media.

### Half-inhibition assay

Half-inhibition assays were carried out according to Vicente-Agullo (2004). After seeds were germinated on half-strength MS medium as described above for 3 d, they were transferred to DEX or EtOH plates and grown vertically for 2 d, then they were transferred to DEX or EtOH plates containing various concentrations of IAA, and the position of root tips was marked. The seedlings were grown for a further 3 d and root elongation during this period was measured. The data were then converted to percentage of growth relative to control elongation on media free of auxin. The half-inhibition concentration (IC_50_) and IC_50_ shift in the dosage response curve was determined through fitting the log (inhibitor) versus response equation and the EC_50_ shift equation in Prism 5.02 (GraphPad Software, CA, USA) to the experimental data.

### GUS staining and observation

GUS staining was done as described previously (Geldner *et al.*, 2004). Roots were collected into ice-cold 90% acetone, and pre-fixed at room temperature for 20min, then washed once with GUS staining buffer [50mM sodium phosphate buffer, 0.2% Triton X-100, 2mM each of K_3_Fe^III^(CN)_6_ and K_4_Fe^II^(CN)_6_] and stained for 2h in GUS staining buffer containing 2mM X-Gluc at 37 °C in the dark. Stained roots were cleared with Hoyer’s solution. Histological observations were performed with an Olympus VANOX microscope equipped with Nomarski optics. Digital images were captured using a MicroPublisher 3.3 RTV cooled CCD camera (Q-imaging, Canada), and processed with Photoshop 8.0 (Adobe).

### Confocal microscopy and image analysis

For observation of GFP fusion protein in root tips, root tips were collected before observation, counterstained with 10 μg ml^–1^ propidium iodide for 1min, and mounted with water on glass slides. GFP fusion protein in LRPs was observed as described (Ditengou *et al.*, 2008); seedlings were fixed with 4% paraformaldehyde in 1× phosphate-buffered saline (PBS) for 2h, and after washing with 1× PBS three times, they were stained with 10 μg l^–1^ propidium iodide overnight at 4 °C. All samples were observed under the Olympus FV1000 confocal microscope using an Olympus UPLAPO ×40 oil-immersion objective (NA=1.00). GFP fluorescence was acquired using excitation with a 488nm laser and a 530nm band pass emission filter. Images were processed with Photoshop 8.0 (Adobe). All images were acquired by the same settings within an independent experiment. The signal intensities of PIN1–GFP on the basal plasma membrane and in the cytoplasm were measured using image J software as described (Du *et al.*, 2013). For each of measured cells, whole basal plasma membrane region was used to quantify GFP intensity of the basal plasma membrane signal, and whole cytoplasm region was used to measure the cytoplasm GFP intensity. The basal plasma membrane/cytoplasm signal ratio of each cell was calculated as the ratio of basal plasma membrane intensity versus cytoplasm intensity.

## Results

### An inducible antisense *GNOM* expression system for manipulating polar auxin transport

**Fig. 1.**
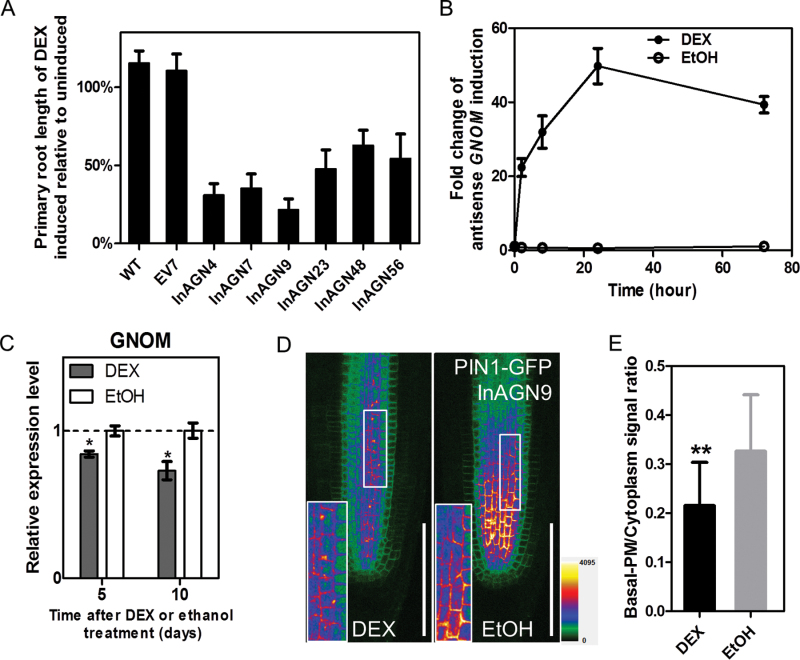
Transient suppression of *GNOM* expression in *Arabidopsis* and phenotypes of seedlings. (A) Primary root length of DEX-induced seedlings relative to uninduced seedlings in different transgenic lines grown on DEX- or EtOH-supplemented MS medium for 7 d after germination. The EV7 line contains empty vector with no antisense construct. The mean ±SE for 24 seedlings in triplicate experiments is plotted. (B) Fold change of the induced *GNOM* antisense transcripts after InAGN9 seedlings were exposed to DEX or EtOH for various times. EtOH indicates the uninduced control. Each data point represents the mean ±SE from three biological repeats and two technical repeats. (C) Expression of endogenous *GNOM* mRNA in induced or uninduced InAGN9 seedlings. Each data point represents the mean ±SE from two biological repeats and two technical repeats. *P<0.05 (Student’s *t*-test). (D) Localization of GFP-fused PIN1 protein in root tip treated with DEX or EtOH. Insets: enlargements of boxed areas, showing details of PIN1 subcellular localization. (E) Fluorescent intensity of PIN1–GFP in the basal plasma membrane relative to that in the cytoplasm. DEX, *n*=30 cells from three roots; EtOH, *n*=52 cells from thee roots; **P<0.01 (Student’s *t*-test). Scale bar=100 μm in (D).

To induce knock-down of *GNOM* expression in *Arabidopsis* roots, transgenic plants (InAGN) containing an antisense *GNOM* fragment were generated in the glucocorticoid-inducible expression vector pTA211 (Supplementary Fig. S1 available at *JXB* online), which allows induction of the antisense mRNA in the presence of DEX (Sanchez and Chua, 2001). By hygromycin resistance screening, 83 transgenic lines were acquired. For each of these lines, the presence of the GAL4 sequence was confirmed by PCR. The T_2_ seeds of these lines were also checked for segregation of hygromycin resistance. All the lines with the presence of GAL4 and a resistance segregation ratio of 3:1 were then selected; 27 independent lines in total. The T_3_ homozygous seedlings of these 27 lines were used for further studies. It was found that DEX treatment induced a variable extent of inhibition of primary root elongation in these 27 transgenic lines. Among them, seven T_3_ lines showed the most significant inhibition of primary root elongation and thus were used for initial induction studies. As a control, a homozygous T_3_ line carrying an empty pTA211 vector (EV7) was also generated. When germinated on half-strength MS medium supplemented with 30 μM DEX (inducer) or 0.1% EtOH (mock) for 7 d, all antisense *GNOM* lines displayed a reduction of primary root length compared with the control lines and mock-treated antisense *GNOM* lines, both of which showed no sign of growth repression (Fig. 1A). Among these seven antisense *GNOM* lines the InAGN9 line showed the shortest length of the primary root and was selected as a representative line for further studies (Fig. 1A).

Quantitative RT–PCR analysis showed that expression of the antisense *GNOM* transcripts in InAGN9 seedlings was markedly induced 2h after DEX application, peaked at 24h after induction, and dropped slightly at 72h, possibly due to degradation of DEX (Fig. 1B). In contrast, only a basal level of expression of antisense *GNOM* transcripts could be detected in mock-treated InAGN9 seedlings (Fig. 1B), indicating that before DEX induction there was no leaky expression of antisense *GNOM* transcripts. Moreover, as expected, it was found that expression of endogenous *GNOM* transcripts was significantly decreased in InAGN9 seedlings after DEX induction compared with the mock control (Fig. 1C). Accordingly, the GNOM protein expression level decreased ~50% in the InAGN9 seedlings compared with that in the mock control (Supplementary Fig. S9 available at *JXB* online), which was checked by western blotting with anti-GNOM antibody (Supplementary Fig. S10 available at *JXB* online). These findings suggest that endogenous GNOM expression could be manipulated using the inducible antisense system. DEX treatment did not change the expression of endogenous mRNA or the protein expression level of *GNOM* gene in EV7 seedlings (Supplementary Figs S2, S9 available at *JXB* online).

GNOM regulates endosomal recycling and transcytosis of PIN1 to the basal plasma membrane (Geldner *et al.*, 2003; Kleine-Vehn *et al.*, 2008). To determine whether PIN1 localization was perturbed, the PIN1–GFP marker was introduced into InAGN9 by crossing the two lines and the subcellular localization of PIN1–GFP was examined before and after DEX induction. No obvious differences in the abundance or localization of PIN1–GFP were observed between InAGN9 and wild-type seedlings grown in the mock conditions (data not shown). However, after transient DEX treatment (4h), accumulation of PIN1–GFP in the basal plasma membrane was markedly reduced (Fig. 1D, E) and the proportion of PIN1–GFP in the endosomal compartment was slightly increased in InAGN9 compared with the mock control (Fig. 1D). This indicated that PIN1–GFP was not efficiently delivered to the basal plasma membrane due to antisense repression of *GNOM* expression. In contrast, it was found that the subcellular localization of other PIN proteins (PIN2, PIN3, and PIN7) was not obviously affected by antisense repression of *GNOM* expression (Supplementary Fig. S3 available at *JXB* online). The retention of PIN1–GFP in the endosomal compartment was observed consistently after treatment with DEX for several days.

### The root elongation zone is most sensitive to the interruption of GNOM-mediated auxin transport

**Fig. 2.**
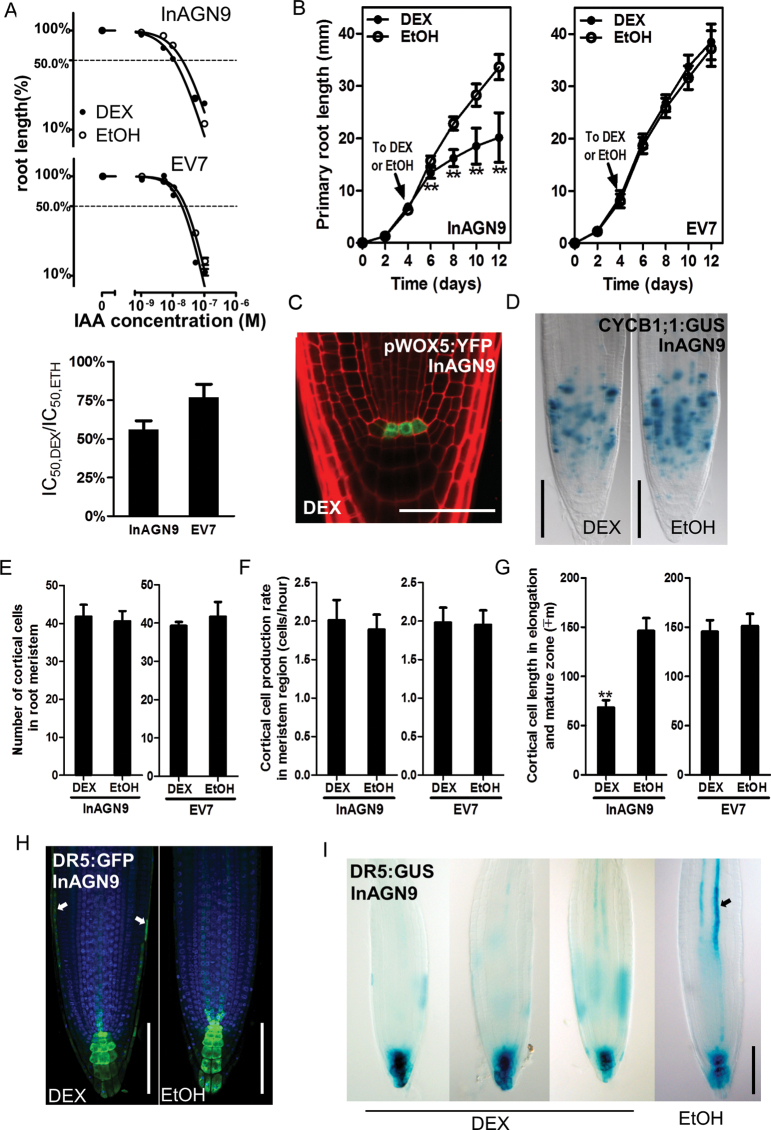
Disruption of auxin transport by DEX induction and the phenotypes of root tips. (A) In the presence of exogenous IAA, root elongation of InAGN9 and EV7 seedlings treated with DEX or EtOH is inhibited in an IAA concentration-dependent manner. EtOH indicates the uninduced control. Relative change of sensitivity to auxin after DEX induction is presented as a shift of the half-inhibition concentration (IC_50_) by calculating the IC_50,DEX_/IC_50,EtOH_ ratio. Each data point represents the mean ±SE of 20–25 seedlings from duplicate experiments. (B) Growth curve of InAGN9 and EV7 roots. Seedlings were transferred to DEX- or EtOH-containing medium at day 4 (indicated by arrow). The mean ±SD for 18–20 seedlings from duplicate experiments is plotted. **P<0.01 (Student’s *t*-test). (C) Expression of pWOX5-YFP in the root tips of DEX-induced InAGN9 seedlings. (D) Expression of CYCB1;1:GUS in the root tips of DEX- or EtOH-treated InAGN9 seedlings. (E–G) Number of meristem cells (E), cell production rate in the cortical cell layer within the meristem zone (F), and cortical cell length in the differentiation zone (G) of DEX- or EtOH-treated InAGN9 seedlings. Data are presented as the mean ±SE from 43–45 seedlings in two or four repeated experiments. **P<0.01 (Student’s *t*-test). (H and I) DR5*rev*::GFP (H) and DR5::GUS (I) expression pattern in DEX- or EtOH-treated InAGN9 seedlings. Arrows in (H) show GFP signals in cells of the lateral root cap; the arrow in (I) shows GUS signal in stele tissue of EtOH-treated root tip. Scale bar=50 μm in (C), 100 μm in (D), (H), and (I).

It was reported that when *Arabidopsis* seeds were germinated on MS medium supplemented with auxin and the elongation of the primary root was inhibited by the exogenous auxin in a concentration-dependent manner (Ivanchenko *et al.*, 2010). The sensitivity of the seedlings to auxin can be estimated by measuring the half-inhibition concentration of auxin (IC_50_), the concentration at which the primary root elongation decreased to half of that in the roots grown on mock medium (Vicente-Agullo *et al.*, 2004). Similarly, the effect of inhibition of *GNOM* on auxin sensitivity was examined by measuring the half-inhibition concentration of exogenous AA (IC_50_). It was found that the IC_50_ in DEX-induced InAGN9 seedlings decreased to half of that in controls (Fig. 2A), indicating that DEX-induced InAGN9 seedlings are more sensitive to IAA. Moreover, when transferred to DEX-containing medium, InAGN9 seedlings showed a reduction in root elongation (Fig. 2B), as previously described for partial loss-of-function *gnom* mutants (Geldner *et al.*, 2004). The expression of the QC marker WOX5::YFP (Sarkar *et al.*, 2007; Fig. 2C) and the cell proliferation marker CYCB1;1::GUS (Colon-Carmona *et al.*, 1999; Fig. 2D) was examined in roots of the treated seedlings and no difference was found in the expression of these two markers between DEX-induced or mock-treated InAGN9 seedlings (Fig. 2C, D). It was also found that the cell numbers (Fig. 2E) and cell proliferation rate (Fig. 2F) of meristematic cortical cells in DEX-induced InAGN9 lines were similar to those in mock-treated lines. These findings suggest that the inducible knock-down of *GNOM* does not lead to defects in the QC and root meristem. The cortical cell length in the elongation and differentiation zones, however, was decreased to half that of cells in mock-treated roots (Fig. 2G), indicating that the root elongation phenotype observed in DEX-treated InAGN9 plants was caused by a reduction in length of elongating and differentiated root cells. These results revealed that, in the root, cell elongation and differentiation, but not cell proliferation are sensitively controlled by GNOM- and PIN1-dependent auxin transport. Consistent with this, expression of the auxin-responsive marker DR5::GUS decreased in the elongating and differentiated vascular cells, compared with that of mock controls (Fig. 2I), and ectopic DR5::GUS and DR5*rev*::GFP expression was observed in lateral root cap cells in addition to the normal distribution pattern (Fig. 2H). This change in the DR5::GUS and DR5*rev*::GFP expression pattern appeared after transient DEX treatment, and longer DEX treatment showed the same effects. Thus, both transient and long-term DEX treatment disrupted auxin transport, as shown by the DR5 response pattern and PIN1 subcellular localization.

### Moderate disruption of GNOM-mediated auxin transport mainly perturbed LRP initiation and root meristem establishment

**Fig. 3.**
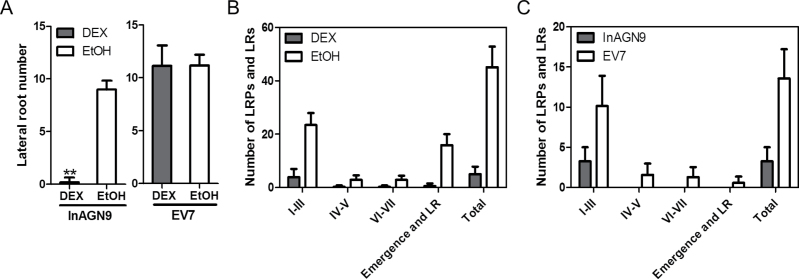
Phenotypes of lateral root formation after disruption of polar auxin transport. (A) Lateral root number of InAGN9 and EV7 seedlings germinated on MS plates containing DEX or EtOH for 10 d. Data represent the mean ±SE from 40–50 seedlings from five repeated experiments. **P<0.01. (B) Number of lateral root primordia and lateral roots in InAGN9 seedlings germinated for 10 d on plates containing DEX or EtOH. Samples were analysed in duplicate and error bars represent the SD (*n*=8). (C) Number of lateral root primordia and lateral roots in InAGN9 and EV7 seedlings germinated for 5 d on plates containing DEX. Samples were analysed in duplicate and error bars represent the SD (*n*=8).

**Fig. 4.**
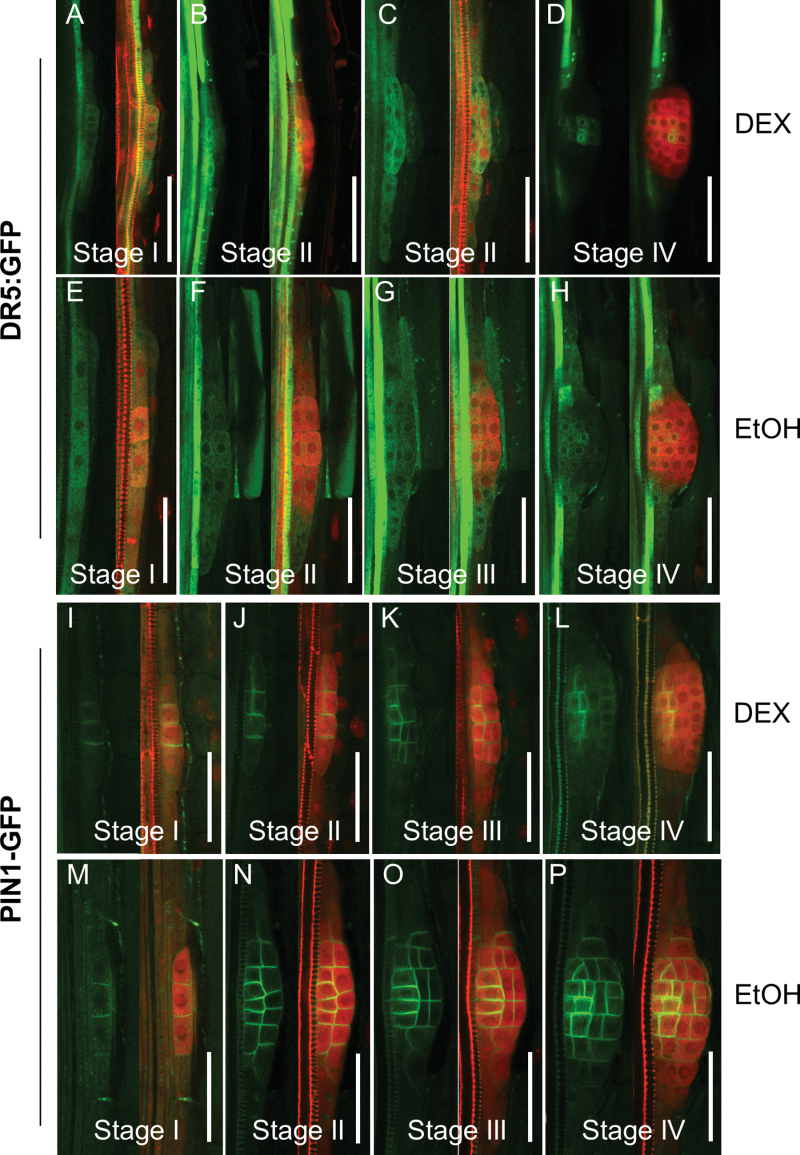
Expression of DR5*rev*::GFP and PIN1–GFP in lateral root primordia in InAGN9 roots treated with DEX or EtOH. (A–H) DR5*rev*::GFP expression (green) in lateral root primordia of DEX- (A–D) and EtOH- (E–H) treated InAGN9 roots. (I–P) Localization of PIN1–GFP (green) in lateral root primordia of DEX- (I–L) or EtOH- (M–P) treated InAGN9 roots. Lateral root primordia were counterstained with propidium iodide (red). Scale bar=50 μm.

It was found that InAGN9 seedlings germinated directly on DEX-containing medium barely formed any lateral roots, whereas mock-treated InAGN9 and EV7 had similar numbers of lateral roots (Fig. 3A). The lateral root phenotype of DEX-treated InAGN9 seedlings resembled what was observed in *gnom* ^*R5*^ mutants (Geldner *et al.*, 2004), except that asymmetric pericycle cell division could still be seen in DEX-treated InAGN9 roots. To understand further at which stage lateral root development ceased, the development of LRPs was examined in InAGN9 and EV7 plants germinated for 5 d or 10 d on DEX-containing or mock medium. The total number of LRPs in DEX-treated InAGN9 roots decreased dramatically compared with the mock controls and DEX-treated EV7 roots (Fig. 3B, C). Most of the LRPs in DEX-treated InAGN9 plants were arrested at stage I–III, whereas in DEX- and mock-treated EV7 seedlings, LRPs initiated and developed properly (Supplementary Fig. S4 available at *JXB* online). These results substantiated a specific role for GNOM in LRP initiation, cell generation, and root meristem establishment, possibly through its function in mediating local auxin gradients. To test this hypothesis, the expression of DR5*rev*::GFP and PIN1–GFP in LRPs was examined upon DEX induction. It was found that most LRPs at stage I (six out of seven LRPs observed in all samples) showed a DR5*rev*::GFP signal (Fig. 4A) similar to the mock control (Fig. 4E). At stage II, five out of 11 LRPs showed strong accumulation of DR5*rev*::GFP in the cells of the inner layer (Fig. 4B) or in peripheral cells instead of in the central region, whereas other stage II LRPs showed a normal DR5*rev*::GFP expression pattern but weaker signal (Fig. 4C) than that of mock controls (Fig. 4F). In larger LRPs, DR5*rev*::GFP was expressed in two files of cells in the central region (Fig. 4D), but not in all the cells of the central region as observed in mock controls (Fig. 4G, H). This observation suggests that GNOM-mediated auxin transport is necessary for the local auxin gradient and thus for both LRP initiation and development. Therefore, these processes are most sensitive to the variation of GNOM-mediated auxin transport.

For PIN1–GFP, it was found that only 50% of observed LRPs (*n*=42) in DEX-treated roots showed GFP signal on the plasma membrane (Fig. 4I), but in mock-treated roots, ~90% of observed LRPs (*n*=64) showed PIN1–GFP expression (Fig. 4M). PIN1–GFP signal in LRPs of DEX-treated roots was lower than in mock-treated roots and showed reduced lateral distribution to newly formed periclinal plasma membrane in LRPs at stage II (Fig. 4J) and later stages (Fig. 4K, L) compared with mock controls (Fig. 4N–P). This indicates that PIN1-dependent auxin transport was indeed interrupted in the LRPs. In contrast, in DEX-treated roots, disorganized cell division was observed in LRPs lacking PIN–GFP expression (Supplementary Fig. S5A–C available at *JXB* online).

**Fig. 5.**
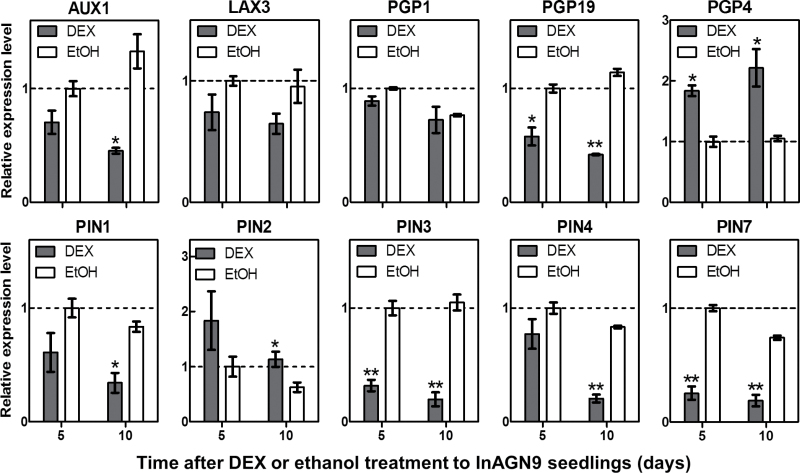
Changes in expression of genes encoding auxin transport proteins after InAGN9 seedlings were treated with DEX. Expression levels of auxin transport genes *AUX1*, *LAX3*, *PGP1*, *PGP19*, *PGP4*, *PIN1*, *PIN2*, *PIN3*, *PIN4*, and *PIN7* measured by quantitative RT–PCR at 5 d or 10 d after induction with DEX (grey bars) or no induction (EtOH, white bars). Levels are measured relative to uninduced controls at 5 d (dashed line). *P<0.05; **P<0.01 (Student’s *t*-test).

Expression of other *PIN* genes was also examined. Quantitative RT–PCR analysis showed reduced expression of *PIN1*, *PIN3*, *PIN4*, and *PIN7*, but increased *PIN2* transcription in DEX-treated InAGN9 lines, compared with the mock control. In addition, expression of several other auxin transporter genes (*PGP19*, *AUX1*, and *LAX3*) was also decreased, whereas *PGP4* expression was up-regulated in DEX-treated seedlings (Fig. 5). In contrast, expression of these genes was not altered in the EV7 line upon DEX induction (Supplementary Fig. S6 available at *JXB* online).

In summary, moderate disruption of GNOM expression disturbed PIN1 relocation and GNOM-dependent auxin transport, and altered expression of *PIN* family genes, resulting in an abnormal auxin sink. LRP initiation and meristem establishment during root generation were notably affected by such gentle modulation, indicating that they are more sensitive than any other developmental processes.

### Disruption of GNOM-mediated auxin transport occurred with reduced expression of auxin biosynthesis genes

**Fig. 6.**
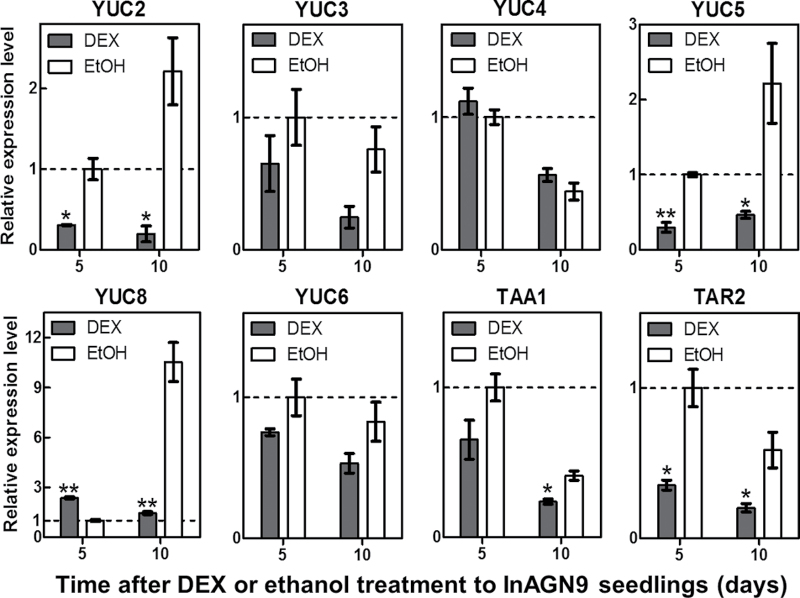
Changes in expression of auxin synthesis genes after InAGN9 seedlings were treated with DEX. Expression levels of auxin synthesis genes *YUC2*, *YUC3*, *YUC4*, *YUC5*, *YUC6*, *YUC8*, *TAA1*, and *TAR2* measured by quantitative RT–PCR at 5 d or 10 d after induction with DEX (grey bars) or no induction (EtOH, white bars). Levels are measured relative to uninduced controls at 5 d (dashed line). * *P*<0.05; **P<0.01 (Student’s *t*-test).

Previous work showed that auxin biosynthesis is also involved in LRP formation (Dubrovsky *et al.*, 2008; Li *et al.*, 2008). The present system provided a unique opportunity to study the connection between auxin transport and auxin biosynthesis during LRP development. Therefore, the expression of genes for auxin biosynthesis was measured after transiently disrupting auxin transport during LRP formation. It was found that expression of many key genes for auxin biosynthesis, including YUCCA family genes (*YUC2*, *YUC3*, *YUC5*, and *YUC6*) and TAA family genes (*TAA1* and *TAR2*) (Won *et al.*, 2011), was down-regulated in DEX-induced seedlings (Fig. 6). The expression levels of these genes did not change in DEX-or mock-treated EV7 seedlings (Supplementary Fig. S7 available at *JXB* online). These results indicate that transient disruption of auxin transport down-regulated major genes for auxin biosynthesis, which probably decrease auxin biosynthesis in response to the disruption of auxin transport.

**Fig. 7.**
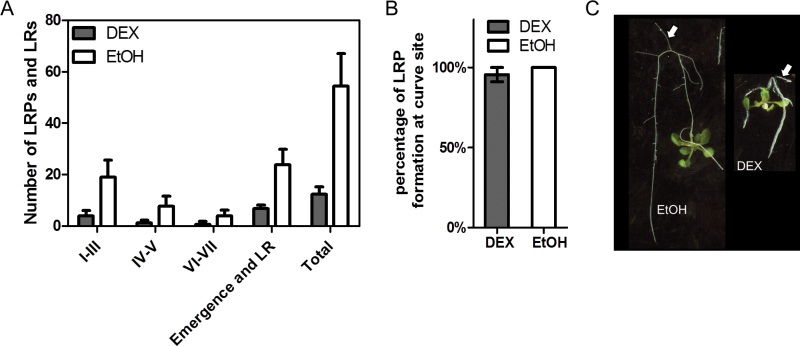
Rescue of lateral root initiation by globally or locally elevating the auxin level. (A) Number of lateral root primordia (LRPs) and lateral roots (LRs) in InAGN9 seedlings that germinated on MS plates containing DEX or EtOH that had been supplemented with 0.1 μM NAA. Data represent the mean±SD (*n*=10). (B) Formation of LRPs at the site of curving of DEX- or EtOH-treated roots. Samples were analysed in triplicate (*n*=15 for EtOH- and *n*=46 for DEX-treated roots). (C) Formation of lateral roots at the site of curving of roots. (This figure is available in colour at *JXB* online.)

An attempt was made to germinate InAGN9 seeds in medium that was supplied with 100nM NAA together with 30 μM DEX, and it was found that the treated InAGN9 seedlings produced more LRPs and a significant amount of LRPs developed into lateral roots. In addition, some LRPs developed beyond stage III and several lateral roots were formed (Fig. 7A). This result indicated that 100nM NAA could partially rescue the defect of lateral root formation in DEX-induced InAGN9 seedlings by promoting the initiation of LRPs and stimulating the primordia to grow into lateral roots.

Manual or gravity-induced bending of the root tip can result in a local increase of auxin in pericycle cells at the outside curvature of the root bending site and eventually induces a lateral root (Ditengou *et al.*, 2008; Laskowski *et al.*, 2008). By a similar technique, DEX-induced InAGN9 roots were bent by turning them 180 ° and leaving them to grow for one more day to form a J-hook, and it was found that LRPs formed at the outside of the root curve as efficiently as in non-induced roots (Fig. 7B). Indeed, 80.8% of DEX-induced roots (*n*=26) successfully produced a lateral root at the curving site and 100% of non-induced roots (*n*=12) produced a lateral root at the curving site after they grew for three more days (Fig. 7C).

The locally elevated auxin levels and re-establishment of the auxin gradient could rescue the defect of lateral root formation in DEX-induced InAGN9 seedlings, indicating that the defects in LRP formation in DEX-induced roots are mainly due to the reduction of the auxin level. This result suggests that decreased expression of major auxin biosynthesis genes probably resulted in the reduction of auxin in LRP initiation sites. Thus, both polar auxin transport and local auxin synthesis may contribute to the maintenance of the auxin levels for normal LRP formation.

## Discussion

### An inducible knock-down system to disrupt GNOM-mediated auxin transport effectively

Using transient knock-down of *GNOM* expression by an inducible antisense expression system, GNOM-mediated auxin transport was successfully disrupted in roots, which was confirmed by changes in the DR5 auxin response marker (Benkova *et al.*, 2003) and the subcellular localization of the PIN1 protein (Xu *et al.*, 2006). This disruption was also evidenced by phenotypes of increased sensitivity to auxin and inhibited root cell elongation after DEX induction. Induced antisense *GNOM* mRNAs were already expressed 2h after DEX induction and peaked after 24h. The abnormal DR5 response pattern appeared 4h after induction and the inhibition of cortical cell elongation could already be observed 12h after induction. Thus, DEX induction can efficiently disrupt GNOM-mediated auxin transport in the established system and result in notable phenotypes. Besides an abnormal DR5 response pattern and disrupted PIN1 localization, the expression of genes that encode proteins transporting auxin in the rootward direction, such as *PIN1*, *PIN3*, *PIN4*, *PIN7*, and *PGP19*, was decreased. However, expression of *PIN2* and *PGP4*, which transport auxin in the shootward direction in the lateral root cap, epidermal, and cortical cells, was increased. These changes in the expression of auxin transport genes indicate that less auxin was transported from stele tissue to the root apex and more auxin was transported out of the apex to the lateral root cap, epidermal and cortical cells, finally resulting in low auxin levels in the meristem region. This was further supported by the presence of extra DR5 auxin response signals in lateral root cap cells, and weak signals in the root meristem region. The inhibited elongation of epidermal and cortical cells in the root elongation and differentiation zone also suggested that the auxin level in those cells was higher than that of normal roots. Thus, the system is capable of effectively disrupting GNOM-mediated auxin transport.

### Tissue-specific response of root to GNOM-mediated auxin transport

**Fig. 8.**
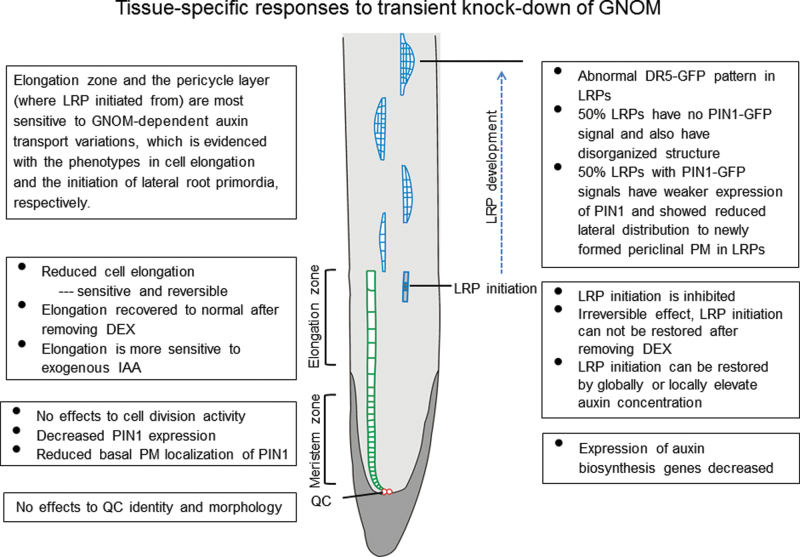
Illustration of all the changes in DEX-treated InAGN9 roots.

After disruption of GNOM-mediated auxin transport, root cells of different types showed different responses (Fig. 8), indicated by their growth behaviour during embryonic root development and lateral root formation. Transient DEX treatment obviously inhibited elongation of the primary root tip. Detailed investigation revealed that the QC cells and meristem region of the root tips were still functional, indicating that the disruption of GNOM-mediated auxin transport did not disrupt root tip meristem maintenance under the moderate modulation. Among the different zones of the root tip, only the elongation zone showed a distinct, abnormal phenotype during disruption of GNOM-mediated auxin transport. Cell elongation in this zone was obviously inhibited. This may reveal the basic nature of the root cell response to auxin dynamics at physiological levels.

Another notable phenotype in DEX-treated InAGN9 root is that no lateral roots were produced, although they could form a few LRPs, which all rested at early stages. This observation indicates that generation of the LRP initiation cell was inhibited and root primordium establishment was also interrupted, showing the specific role of GNOM-mediated auxin transport in these processes. This is consistent with the recent study on a novel weak *gnom* allele, *fwr*, which also showed that LRP initiation is sensitive to GNOM activity (Okumura *et al.*, 2013). In fact, tissue-specific expression of *GNOM* clearly showed that provascular expression of *GNOM* in the early embryo was required for the establishment of the root meristem and probably for maintenance of the meristem (Wolters *et al.*, 2011). In the present work, it was further confirmed that GNOM-mediated auxin transport is required for LRP establishment.

Based on experiments with tissue-specific expression of *GNOM*, Wolters *et al.* (2011) proposed that GNOM-dependent auxin sinks could generate auxin gradients across tissues in the embryo. In the present work, the abnormal DR5 pattern after DEX treatment during LRP generation and development suggested that the local auxin gradient was interrupted, probably because the GNOM-dependent auxin sink was not correctly established. This is consistent with the idea that GNOM-dependent auxin drainage is vital for meristem establishment in the embryo. This work provides an example of how the GNOM-dependent auxin sink and its generated auxin gradient could regulate cell growth and differentiation of specific tissues.

Together, these results indicate that root development responds to GNOM-dependent auxin transport in a tissue-specific manner and the most sensitive areas are the root tip elongation zone for cell elongation and pericycle cells for their transition to LRP initiation cells. The QC and meristem region possess a great potential to maintain their cell nature during moderate auxin variations. The present data suggest that the cells during differentiation or cell fate transition are much more sensitive to auxin dynamics.

### GNOM-mediated auxin transport and auxin biosynthesis are related and may have joint effects on lateral root initiation and development

The important role of auxin during root development has been extensively studied (Casimiro *et al.*, 2001; Bhalerao *et al.*, 2002; Himanen *et al.*, 2002; Benkova *et al.*, 2003; Cheng *et al.*, 2007; De Smet *et al.*, 2007; Grieneisen *et al.*, 2007; Ditengou *et al.*, 2008; Dubrovsky *et al.*, 2008; Laskowski *et al.*, 2008; Zhao, 2008). It has been proposed that both auxin transport and auxin synthesis produce a threshold auxin level, which is required for initiation of organs (Zhao, 2008). Here, evidence is provided of how GNOM-mediated auxin transport relates to auxin biosynthesis and functions during lateral root formation.

After disruption of GNOM-mediated auxin transport, expression of key genes for local auxin biosynthesis also significantly decreased, which implies that local auxin synthesis is probably decreased. The findings suggest that DEX induction reduced auxin levels in the meristem region by disrupting GNOM-mediated auxin transport and possibly also by reducing local auxin biosynthesis, which inhibited LRP initiation. This is further supported by the rescue of LRP initiation through a globally and locally elevated auxin level in DEX-induced InAGN9 roots. Thus, GNOM-mediated auxin transport and auxin biosynthesis have a joint effect on initiation of LRPs, which is consistent with the hypothesis that both auxin transport and auxin biosynthesis produced a threshold level of auxin for initiation of organs (Zhao, 2008). Therefore, it is possible that auxin biosynthesis also contributed to GNOM-dependent auxin sinks during meristem organization (Wolters *et al.*, 2011).

Besides rescuing LRP initiation, elevated auxin levels also promoted LRPs to develop further into lateral roots. Previous studies showed that exogenous auxin can only initiate uniform proliferation of pericycle cells in mutants defective in auxin transport, which could not form normal LRPs (Benkova *et al.*, 2003; Geldner *et al.*, 2004). However, in the present work, globally elevated auxin levels partially rescued defects in lateral root initiation by DEX induction, and LRPs with normal morphology were formed. Therefore, it is believed that DEX induction only partially disrupted auxin transport, and it could still function as a buffer system to maintain proper auxin gradients under globally elevated auxin levels. This was further supported by the appearance of focused DR5 auxin response signals in the QC cells of root tips and in some of the LRPs of DEX-induced InAGN9 seedlings. It was also supported by the proper localization of PIN1 in induced root tips and LRPs although its expression was down-regulated. In mutants with partial defects in auxin transport, such as single mutants of *PIN* genes or *AUX1*, formation of lateral roots was only slightly disrupted (Benkova *et al.*, 2003) or positioning of lateral roots was changed (De Smet *et al.*, 2007). Thus, the disrupted GNOM-mediated auxin transport in DEX-induced InAGN9 seedlings could not be the sole reason for the defects in LRP development. Since expression of most genes related to local auxin synthesis decreased after DEX induction, it was inferred that decreased local auxin biosynthesis also accounts for auxin deficiency at the LRP sites. This speculation is supported by experiments of both global and local elevation of auxin levels in DEX-induced InAGN9 roots, which could rescue the defect of LRP development in these roots. Therefore, auxin biosynthesis and auxin transport both play important roles in LRP development. This idea is consistent with the results of Laskowski *et al.* (1995) that LRPs of one cell layer in an excised 0.5mm root segment could develop 2–3 layered LRPs after being cultured for 7 d. Without auxin transported from seedlings, in this case the excised single-layered LRPs should have synthesized auxin to maintain their own development. However, without transported auxin, the auxin synthesized by early LRPs might not be sufficient for their continued development.

A previous mutant study showed that auxin transport and auxin biosynthesis have synergistic genetic effects (Cheng *et al.*, 2007). However, their relationship has remained largely unclear. Auxin is constantly refluxed within the root tip by polar auxin transport, which maintains the proper gradient (Blilou *et al.*, 2005), while all root cells can synthesize auxin (Petersson *et al.*, 2009). It was also reported that genes of both auxin biosynthesis and transport could be regulated by a common family of transcription factor, INDETERMINATE DOMAIN (IDD) transcription factor. IDD14, IDD15, and IDD16 could regulate lateral organ morphogenesis and gravitropism by targeting the auxin biosynthesis genes *YUC5* and T*AA1*, and the auxin transport gene *PIN1* to promote auxin biosynthesis and transport (Cui *et al.*, 2013). In fact, auxin can regulate its own transport by modulating PIN family proteins both at the transcriptional level (Vieten *et al.*, 2005) and by affecting vesicle trafficking required for proper PIN protein subcellular localization (Paciorek *et al.*, 2005). Therefore, auxin synthesis can indirectly affect auxin transport to a certain degree. There have been few studies concerning the effect on auxin synthesis in auxin transport-defective mutants. The influence of auxin transport on auxin synthesis has remained unclear. In this study, disruption of auxin transport by down-regulating *GNOM* expression decreased the expression level of auxin biosynthesis genes and probably decreased the auxin concentration in the sites of LRP initiation, showing that auxin transport may sensitively affect auxin synthesis by an unknown pathway. A similar phenotype was also recently observed in the weak *gnom* allele *fwr* mutant that fails to form an auxin maximum at LRP initiation sites and also inhibits lateral root formation. Interestingly, the defects could also be restored by exogenously applying auxin (Okumura *et al.*, 2013). In addition, Petersson *et al.* (2009) reported that root tip cells have a high capacity for local auxin synthesis and provide an important IAA source for root tip growth. These data support the present idea that local auxin biosynthesis and auxin transport could work together to maintain the auxin gradient and auxin maximum at the root tip.

Taken together, the present results indicate that auxin transport and biosynthesis are interconnected, and both auxin transport and auxin synthesis may work together to produce a threshold auxin level for LRP initiation and development.

## Supplementary data

Supplementary data are available at *JXB* online.

Figure S1. Schematic of inducible antisense vector construction.

Figure S2. Expression of endogenous *GNOM* mRNA in EV7 seedlings treated with DEX or EtOH for the indicated time.

Figure S3. Expression and localization of other PIN family proteins in DEX- or EtOH-treated InAGN9 roots.

Figure S4. Number of lateral root primordia and lateral roots in EV7 seedlings germinated on plates containing DEX or EtOH.

Figure S5. PIN1–GFP expression in three disorganized LRPs of DEX-induced InAGN9 roots.

Figure S6. Quantitative analysis of changes in expression level of genes encoding auxin transport proteins after EV7 seedlings were induced by DEX.

Figure S7. Quantitative analysis of changes in expression level of auxin synthesis genes after EV7 seedlings were induced by DEX.

Figure S8. Number of lateral root primordia and lateral roots in EV7 seedlings that germinated on MS plates containing DEX or EtOH that has been supplemented with 0.1 μM NAA.

Figure S9. Detection of the GNOM protein level in DEX-treated InAGN9 seedlings by western blotting.

Figure S10. Production of rabbit anti-GNOM polyclonal antibody.

Table S1. Sequence of reverse tramscription primers.

Table S2. Sequence of primers for quantitative PCR.

Supplementary Data
