# Supplementary material for: Inducible knock-down of GNOM during root formation reveals tissue-specific response to auxin transport and its modulation of local auxin biosynthesis
Source: J Exp Bot. 2014 Jan 22;65(4):1165–79. doi: 10.1093/jxb/ert475 (PMC3935571; doi:10.1093/jxb/ert475)
Supplement: Supplementary Data [file supp_65_4_1165__index.html]

Inducible knock-down of GNOM during root formation reveals tissue-specific response to auxin transport and its modulation of local auxin biosynthesis — Inducible knock-down of GNOM during root formation reveals tissue-specific response to auxin transport and its modulation of local auxin biosynthesis — Supplementary Data 

# Inducible knock-down of *GNOM* during root formation reveals tissue-specific response to auxin transport and its modulation of local auxin biosynthesis

## Supplementary Data

Data files

**Files in this Data Supplement:**

- Supplementary Data - Supplementary Data
