# Supplementary material for: Inducible knock-down of GNOM during root formation reveals tissue-specific response to auxin transport and its modulation of local auxin biosynthesis
Source: J Exp Bot. 2014 Jan 22;65(4):1165–79. doi: 10.1093/jxb/ert475 (PMC3935571; doi:10.1093/jxb/ert475)
Supplement: Supplementary Data [file supp_ert475_jexbot112813_file001.pdf]

Inducible knock-down of GNOM during root formation reveals tissue-specific response to auxin transport and its modulation of local auxin biosynthesis

Jingze Guo, Jun Wei, Jian Xu, and Meng-xiang Sun

SUPPLEMENTARY MATERIAL

Supplementary Table S1. Sequence of RT primers

At5g25760	*UBC*	TATTCACTCTCGCAAATCAA
At1g13320	*PP2A subunit A3*	TACTCTCCAGTGCCTGTCTT
At4g26410	*Expressed unknown protein*	TGCCGAAGTCCGTCCATC
At4g34270	*TIP41 like*	CTGGAAGCCTCTGACTGATG
At5g46630	*Clathrin adaptor complex subunit*	TGTTGTAACCGCTCTTCTCC
	Induced antisense *GNOM*	AGCGGTTATGAGAAGAAATGTAAG

Supplementary Table S2. Sequence of primers for quantitative PCR

locus number	gene	primer sequences	product size	primer design
AT1G13320	*PROTEIN PHOSPHATASE 2A SUBUNIT A3*	GTCCTGGCGTGTGCGTTATATG	90 bp	J [b]
		GGCACCAGATCCGTC^CTAGTTG [a]		
AT4G26410	*Expressed unknown protein*	GATTGGTGTCGCTGCTAGTCTC	98 bp	J
		AGAATTGTGC^CTCTTCGCTCTG		
AT4G34270	*TIP41 like*	CTGAGAGTTGATGGTGTGCTTATGAG	141 bp	J
		TGGATACCCTTT^CGCAGATAGAGAC		
AT5G25760	*UBC*	CTGCGACTCAG^GGAATCTTCTAA	61 bp	J
		TTGTGCCATTGAATTGAACCC		
AT5G46630	*Clathrin adaptor complex subunit*	CCAAGACCAATTTCCAAGTGACAAC	78 bp	J
		TTCTTATC^TTCCAAACCAAGCAATCG		
At1g73590	*PIN1*	CTGGTCCCTCATTTCCTTCAA^GTG	116 bp	J
		TGAACAACC^CAAGACTGAACATAGC		
At5g57090	*PIN2*	TCCTCCATATCGCCATCGTTCAG	118 bp	J
		AGCATTCCGAATATAACC^GCAGTG		
At1g70940	*PIN3*	ATGGCGGTTAGGTTCCTTACGG	134 bp	S [c]
		GCAAACACAAAGGGCACAATTCC		
At2g01420	*PIN4*	ATCATTGCTTGTGGGAACTCTGTC	141 bp	J
		CAACGCAGC^CTGAACGATGG		
At1g23080	*PIN7*	AGTGTGATGACTCGGCTGATATTG	114 bp	J
		ATCCCAC^CTGAAAGCAACAAGAG		
At2g36910	*PGP1*	CCGTTATGATTGGTGGATT^GGCATTG	134 bp	J
		GGACTCGCTGTTACGCTCTATCG		
At2g47000	*PGP4*	GCTTCTCTTTCTTCGTCCTCTTCTC	138 bp	S
		ATATCGCCATTGCCGCCATTG		
At3g28860	*PGP19*	TTCTCTGCTATTGTTGGTGGAAT^GAG	130 bp	J
		GGTCTTGAATTATCGTCGGTCTCTG		
At2g38120	*AUX1*	TGCCTCCGCTCGTCAG^AATG	122 bp	J
		CACCGAACCCAAATCCGACTATAAG		
At1g77690	*LAX3*	GCCGTCACAGT^GGAGATAATGC	102 bp	J
		GGATGGTAGCGTTAGCGTTAGTAC		
At4g13260	*YUCCA2*	ACTTCAATGCTCTTCCTTCTCTTGTG	188 bp	S
		AACTAACCCTAACCGATCCGTGTC		
At1g04610	*YUCCA3*	AATCATAAAGTTCGGCAAAGGCAAAG	137 bp	S
		CATCATCGGAGAAGAAGTCGTTGTC		
At5g11320	*YUCCA4*	CGTTCTTGATGTCGGTGCCATTTC	60 bp	J
		GCTTGCGTCACTTTAA^TTTGTCCTG		
At5g43890	*YUCCA5*	GGCGAGAAATACAGAGGAAAGAGTG	125 bp	Without

		ACATGAACCGAGCTACGAACAAC		intron
At5g25620	*YUCCA6*	AACTTCGGTGCTCAGCCTTCTC	68 bp	S
		CCCAACATCTCTCGTGGTAGGAC		
At4g28720	*YUCCA8*	GGATTGTATGCGGTTGGGTTTACG	83 bp	Without intron
		GAGCCTATGTCTTGTGCGATCTTAAC		
At1g70560	*TAA1*	CAGACGACGACGAAGCCAAAG	152 bp	J
		CACCAATGCCCAC^CCAATACG		
At4g24670	*TAR2*	TGTGGGAATGTAACGGGCAAATC	172 bp	J
		CAAATG^CTGGTTGTGGCTCAAAG		
At4g31500	*CYP83B1*	GACCAACCTTTCTCCATCAAATTCAC	176 bp	S
		TCACCTATCACACTCCTCACTTCG		
At4g39950	*CYP79B2*	TGCTTACCGCCGATGAAATCAAAC	165 bp	S
		GTCTCTCTTTCCCGACGACTCTG		
At2g22330	*CYP79B3*	CCTTTGCTTACCGCTGATGAAATC	78 bp	S
		GGCGTTTGATGGGTTGTCTGG		
At2g20610	*SUR1*	AGGCATATCTAAGGGATGGGTTGTTC	143 bp	S
		TTATTGTGGCAGGGTCAGGAGTTAC		
At1g13980	*GNOM*	CTGGTAGCATCAAACAAGAG^AAAGC	62 bp	J
		TGGTTTACTCACAATGGCATAATCAC		

[a] ^ positioned exon-exon junction, where this primer is bridged.
[b] J indicates that the primer was designed to bridge exon-exon junction.
[c] S indicates that the primer pair spanned an intron.

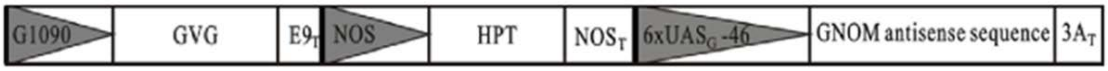

Supplementary Figure S1. Schematic illustration of inducible antisense vector construction

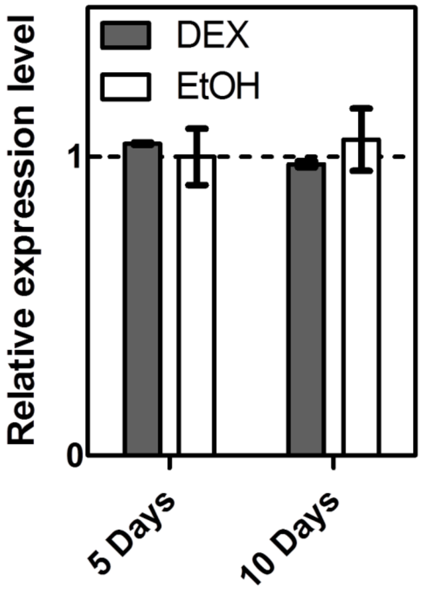

Supplementalry Figure S2. Expression of endogenous *GNOM* mRNA in EV7 seedlings treated with DEX or EtOH by indicated time. Each data point represents mean ± SE from two biological repeats and two technical repeats.

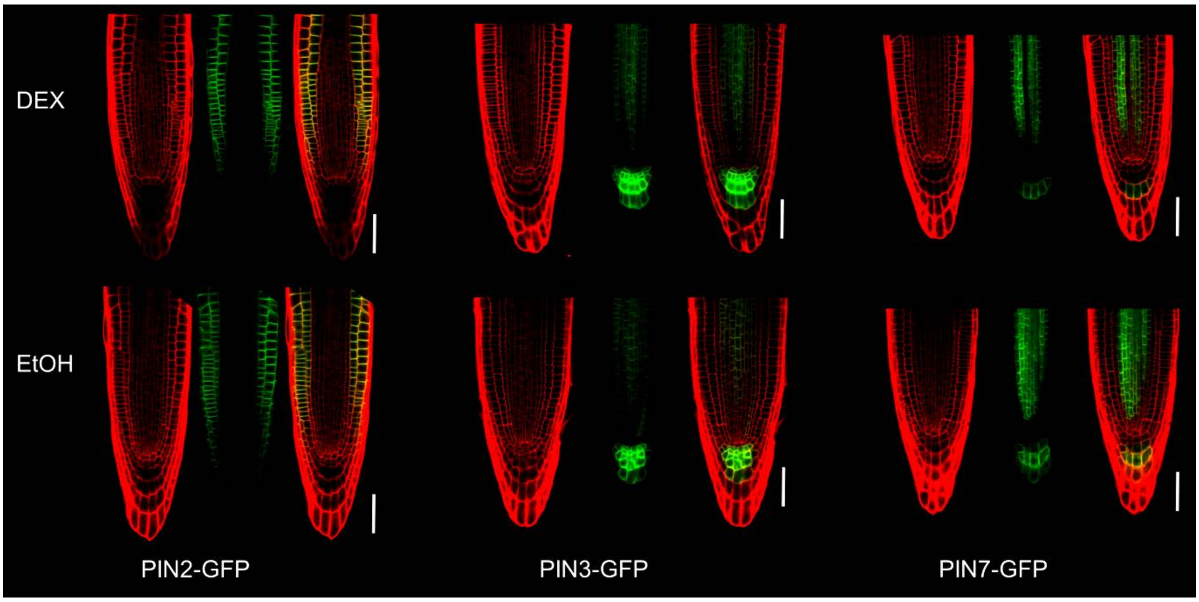

Supplementary Figure S3. Expression and localization of other PIN family proteins in DEX or EtOH treated InAGN9 roots. Roots were counterstained with PI (red). Scale bar: 50 μm

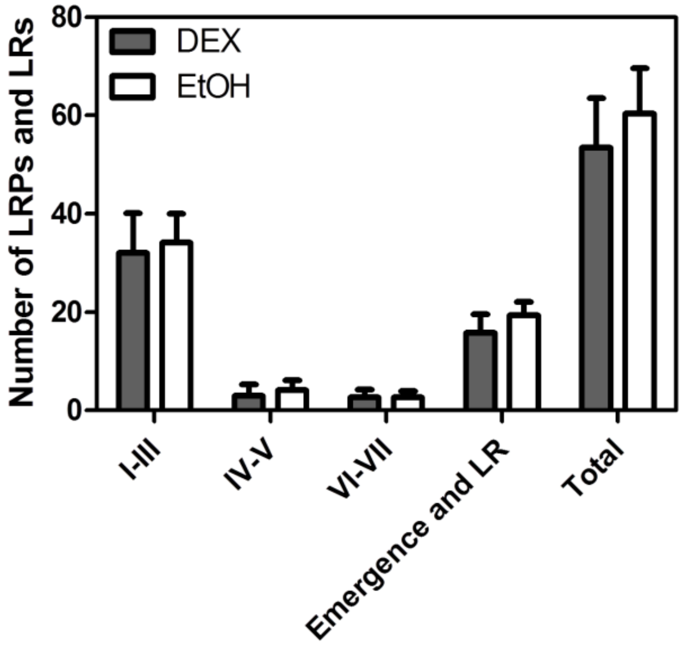

Supplementary Figure S4. Number of lateral root primordia and lateral roots in EV7 seedlings germinated on plates containing DEX or EtOH.

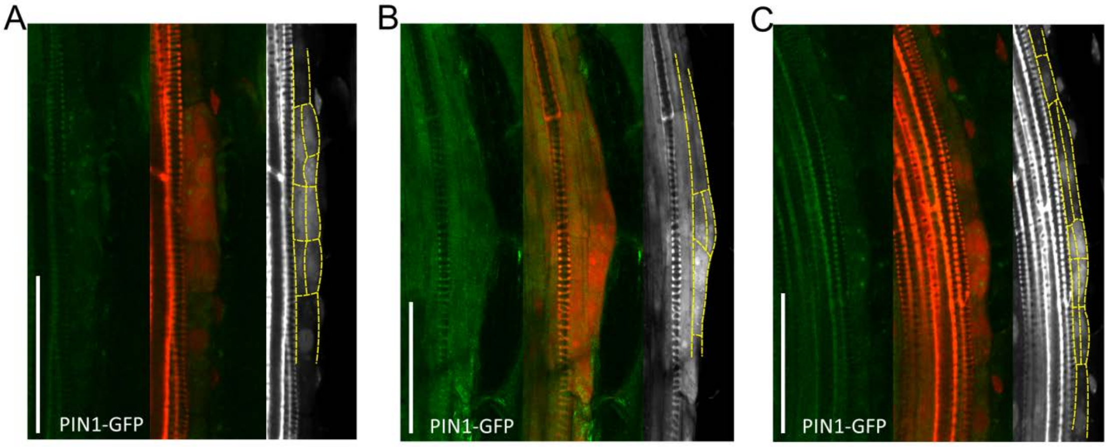

Supplementary Figure S5.PIN1-GFP expression in three disorganized LRPs of DEX induced InAGN9 roots.A-C showed three disorganized LRPs without PIN1-GFP expression. Yellow dotted lines delineate shape of cells in these LRPs. Scale bar: 50 μm

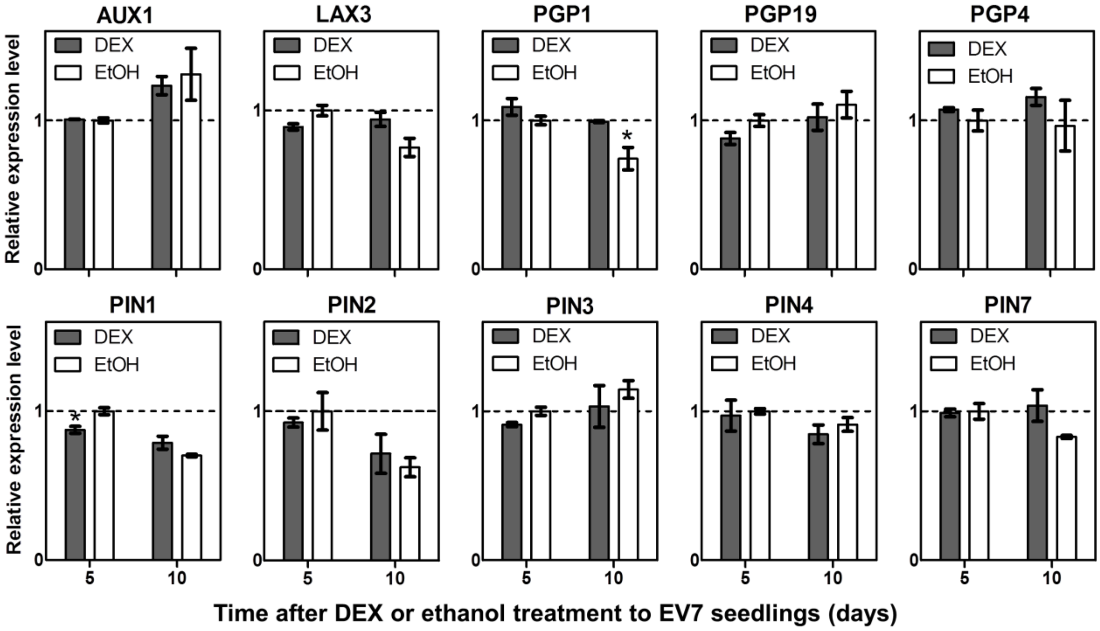

Supplementary Figure S6. Quantitative analysis of changes in expression level of genes encoding auxin transport proteins after EV7 seedlings were induced by DEX.

* indicates $p<0.05$ (Students's *t*-test)

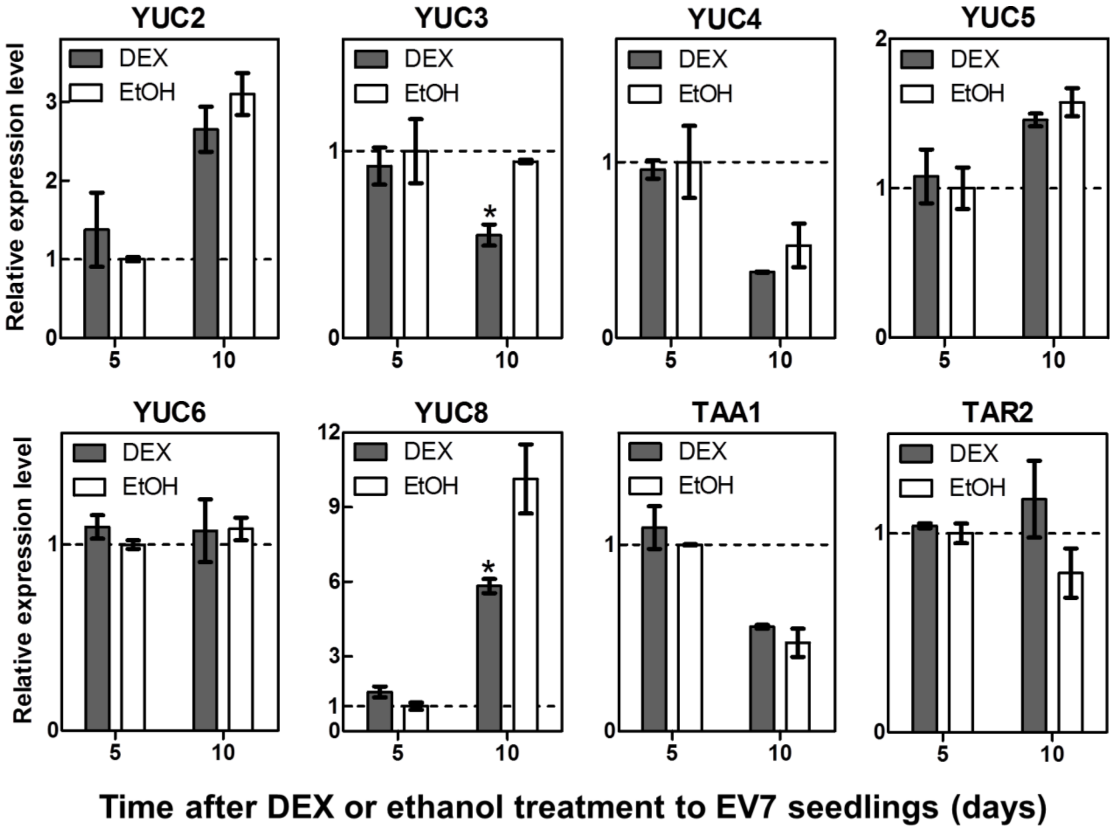

Supplementary Figure S7. Quantitative analysis of changes in expression level of auxin synthesis genes after EV7 seedlings were induced by DEX.

* indicates p<0.05 (Students's*t*-test)

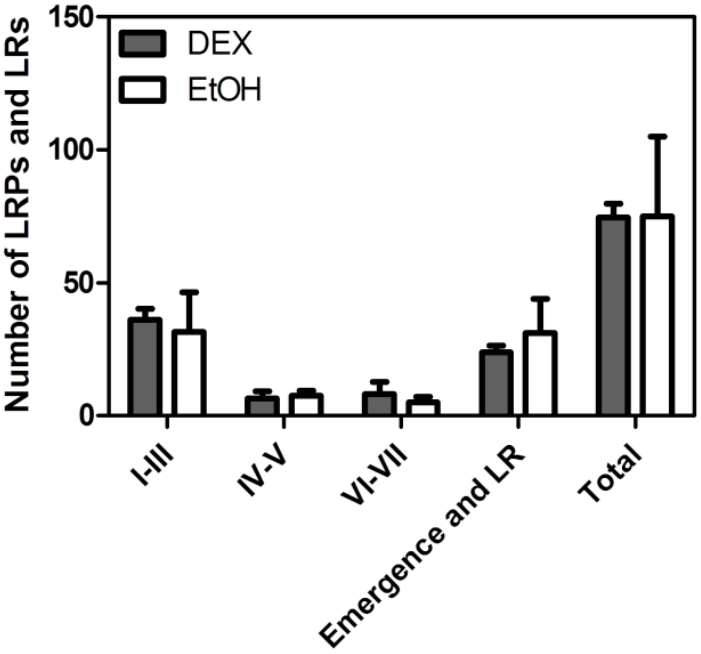

Supplementary Figure S8. Number of lateral root primordia and lateral roots in EV7 seedlings grown on MS plates containing DEX or EtOH with 0.1 μM NAA. Data represent mean ± SD (n=10).

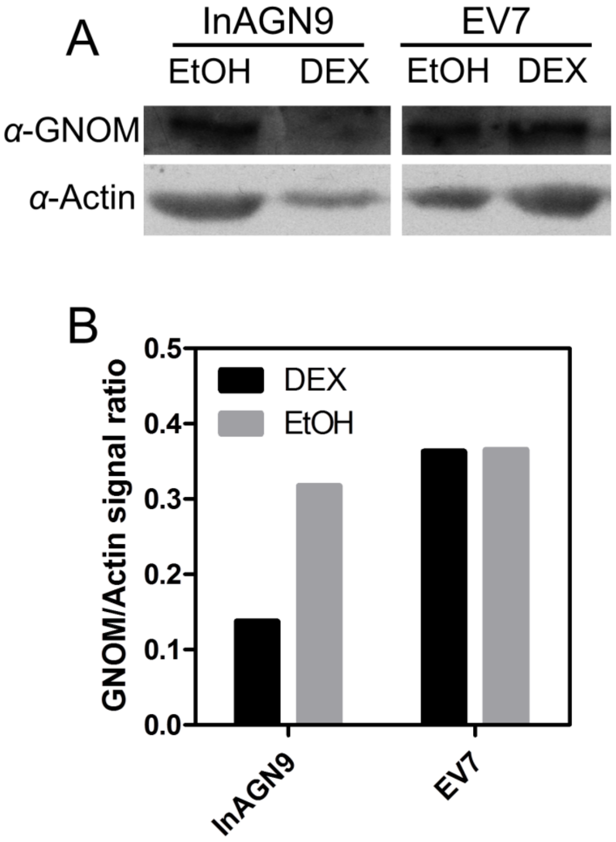

Supplementary Figure S9.Detection of GNOM protein level in DEX treated InAGN9 and EV7 seedlings by western blotting. (A) Total protein was extracted from InAGN9 and EV7 whole seedlings that germinated for 7 days on DEX or EtOH containing MS medium, then they were used for western blotting to detect protein expression level of GNOM and β-ACTINwith anti-GNOM and anti-β-ACTIN (Santa Cruz, USA) antibodies as described [1]. **(B)** Quantification of signal intensity for GNOM and β-ACTIN expression level using Image J software (rsbweb.nih.gov/ij/). Relative protein expression level of GNOM to β-ACTIN was calculated as the ratio of signal intensity of GNOM to β-ACTIN.

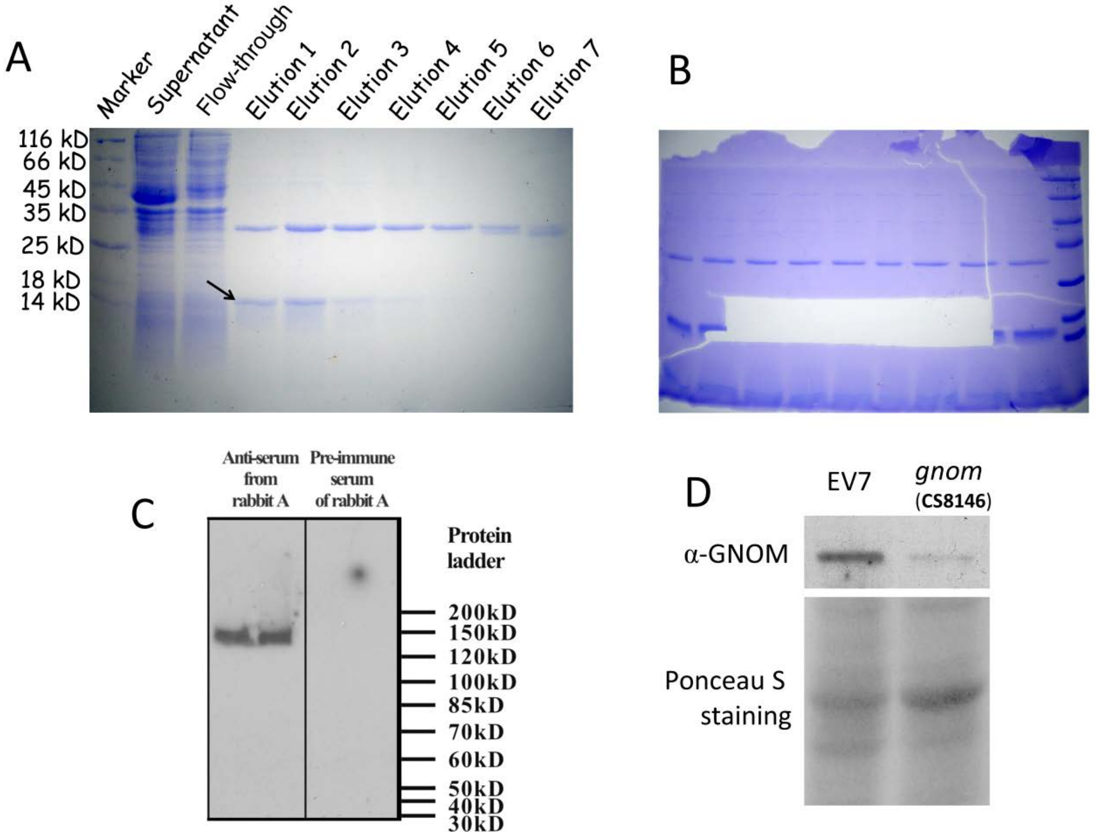

Supplementary Figure S10.Production of rabbit anti-GNOM polyclonal antibody.

(A) Expression and purification of GNOM fragment. Arrow indicated purified GNOM protein fragment. For construction of expression vector pMXB10-GNexp, 1734bp to 2198bp of GNOM coding sequence was amplified from a whole seedling cDNA library and was cloned in-frame into pMXB10 (NEB) using Nde I and Xho I sites. The plasmid was transformed into E. coli. Strain BL21 (DE3) and recombinant protein was induced and purified according to manual of IMPACT system (NEB, https://www.neb.com/~/media/Catalog/All-Products/21A73B351DD24F94BC584FAED2A83A0F/Datacards%20or%20Manuals/manualE6901.pdf). **(B)** Target protein was cut from the polyacrylamide gel after staining with CuCl2, the cutting was confirmed after staining the remaining part of gel by Coomassie blue R250 as described [1].The cut gel with target protein fragment was used to produce antibody in rabbits as described [1]. **(C)** Produced antiserum in rabbit could detect a single band (approximate 150kDa) in proteins from wild type Arabidopsis seedlings, while the pre-immune serum failed to detect any signals. **(D)** The

specificity of the antiserum was confirmed using proteins from a*gnom* mutant (ABRC number: CS8146. As described by Busch [2], this EMS-mutagenized mutant has a point mutation, which produce a premature stop codon at AA647.), because it failed to detect a strong 150kDa band in the mutant protein compared with that of EV7 transgenic line.

References

1. Wang, L., et al., *NtGNL1 is involved in embryonic cell division patterning, root elongation, and pollen tube growth in tobacco.* New Phytol, 2008. **179**(1): p. 81-93.
2. Busch, M., U. Mayer, and G. Jurgens, *Molecular analysis of the Arabidopsis pattern formation of gene GNOM: gene structure and intragenic complementation.* Mol Gen Genet, 1996. **250**(6): p. 681-91.
